# Dermatologic Manifestations of Endocrine Disorders

**DOI:** 10.7759/cureus.18327

**Published:** 2021-09-27

**Authors:** Rishi Raj, Ghada Elshimy, Rahul Mishra, Nivedita Jha, Vismaya Joseph, Russell Bratman, Sri Harsha Tella, Ricardo Correa

**Affiliations:** 1 Endocrinology, Diabetes and Metabolism, Pikeville Medical Center, Pikeville, USA; 2 Endocrinology, Diabetes and Metabolism, Augusta University Medical College of Georgia, Augusta, USA; 3 Internal Medicine, Maulana Azad Medical College, Delhi, IND; 4 Internal Medicine, Adichunchanagiri Institute of Medical Sciences, Karnataka, IND; 5 Data Science, University of Texas at Dallas, Richardson, USA; 6 Endocrinology, Diabetes and Metabolism, Brown University, Rhode Island, USA; 7 Medical Oncology, Mayo Clinic, Rochester, USA; 8 Endocrinology, Diabetes and Metabolism, University of Arizona College of Medicine – Phoenix, Phoenix, USA

**Keywords:** skin lesion, endocrine disorders, dermatologic manifestation, clinical dermatology, endocrine

## Abstract

Endocrine disorders are known to involve all organ systems of the body, including the skin. The cutaneous manifestations of endocrine disorders can range from common findings such as acanthosis nigricans, pretibial myxedema, acne, hirsutism, hyper or hypopigmentation to rare cutaneous findings such as miliaria rubra, calciphylaxis, lentigines, and calcinosis cutis. These cutaneous symptoms can sometimes be the presenting symptoms or can even be pathognomonic of the underlying endocrine condition. In some cases, the cutaneous symptoms from an underlying endocrine abnormality may be the most prominent. These symptoms can significantly affect the quality of life of individuals. Often, individuals may seek health care from a dermatologist or primary care physician for isolated skin symptoms. Therefore, it is imperative for physicians to recognize the skin symptoms as the manifestation of the endocrine disorder for prompt diagnosis and treatment of the underlying endocrine disorder.

## Introduction and background

Hormones are critical regulators of several physiological processes in the human body and play an important role in each system. Their deficiency or excess, as seen in endocrine disorders, can result in numerous dermatologic manifestations. The dermatologic manifestations seen in endocrine disorders can range from mild to severe forms and may or may not be specific. Some skin manifestations may be early in onset and can help in the early diagnosis and treatment of the underlying endocrine disorder. Whenever possible, it is crucial to identify the underlying endocrine disorder to provide corrective treatment for skin symptoms rather than providing symptomatic treatment. In this paper, we present a comprehensive review of up-to-date literature on endocrinopathies and their associated skin conditions and summarize the key findings.

## Review

Graves’ disease

Graves’ disease is an autoimmune thyroid disorder, characterized by the presence of autoantibodies to the thyrotropin receptors (TRAb), which stimulates TRAb and leads to clinical manifestations of hyperthyroidism. While some of the cutaneous manifestations seen among patients with Graves’ disease are due to excess thyroid hormone, certain extrathyroidal manifestations occur due to the effect of thyroid-stimulating immunoglobulins on TRAb found in the skin and connective tissue.

Skin Manifestations

Excess thyroid hormones lead to the overstimulation of adrenergic receptors, leading to increased body temperature and hyperhidrosis, which dermatologically manifests as soft, warm, and moist skin. Hyperhidrosis is typically observed in palms and soles. Loosening of the nails from the nail bed, also known as onycholysis or Plummer’s nails, can also be seen [1]. Pretibial myxedema, also known as thyroid dermopathy, presents as flesh-colored or yellowish-brown, raised skin lesions overlying a nonpitting thickening and induration. The most common site of pretibial myxedema is the anterior tibia followed by the dorsum of the feet and the toes [2,3]. Areas subjected to repeated trauma, surgical scars, and burns are also prone to develop these lesions. In more advanced stages, these lesions may become hyperkeratotic, nodular, and/or fungating [3]. In addition, thyroid dermopathy may manifest as nonpitting lower extremity edema, which in its extreme form presents as elephantiasis of the lower extremity related to the obstruction of lymphatic drainage [3]. Approximately 20% of patients with thyroid dermopathy exhibit acropathy (clubbing of the digits), which is a rare manifestation of Graves’ disease [2]. Furthermore, coexisting Graves’ ophthalmopathy is seen in 96% of cases. While there are case reports of isolated thyroid dermopathy, it is extremely uncommon in the absence of other clinical manifestations of Graves’ disease [3]. Palmar telangiectasias have also been described in a patient with Graves’ disease [4].

Hashimoto’s thyroiditis

In the United States, the most common etiology for the development of hypothyroidism is Hashimoto’s thyroiditis or chronic lymphocytic thyroiditis. It is one of the most commonly encountered endocrine disorders in clinical practice characterized by the reduced production of thyroid hormones.

Skin Manifestations

Reduced response to adrenergic activity in patients with hypothyroidism results in a low metabolic rate which ultimately leads to cold and dry skin. In severe cases of hypothyroidism, the accumulation of mucopolysaccharides leads to a classical presentation called myxedema. The pretibial area is the most commonly involved site [1,5]. In more advanced stages of myxedema, development of macroglossia, broadening of the nose and thickening of the lips, erythematous skin eruption mimicking dermatomyositis [5], and erythema annulare centrifugum [6] have been reported in patients with Hashimoto’s thyroiditis [7].

Glucagonoma

Glucagonoma is a malignant tumor of the alpha cell of the pancreas which secretes the hormone glucagon. The glucagonoma syndrome is characterized by the triad of glucagonoma, diabetes mellitus, and a skin lesion known as necrolytic migratory erythema (NME). Diagnosis requires evidence of an elevated glucagon level, a pancreatic tumor, and clinical manifestations of the disease. Manifestations of glucagonoma may also include weight loss, anemia, and venous thromboembolic disease, as well as gastrointestinal and psychiatric manifestations [8].

Skin Manifestations

NME is the key dermatologic manifestation of glucagonoma [8,9], occurring in nearly all patients during the course of the disease, although it may not be present at the time of presentation. The initial dermatologic eruption of NME is characterized by erythema that progresses to form painful, pruritic bullae and/or vesicular lesions over the course of the next seven to fourteen days. These lesions may then unroof or rupture and spread outward, frequently with hyperpigmentation and crusting of the affected areas [8,9]. Although NME may be widespread, it tends to form in the groin and then spread to the extremities, thighs, buttocks, and perineum. These lesions are often pruritic and can be painful. The perioral area may also be affected. Superinfection, either bacterial or fungal, is common. In areas exposed to constant friction, NME lesions may become lichenified or resemble psoriasis. These lesions may also occur in areas exposed to trauma, a pattern known as koebnerization. Angular cheilitis and/or stomatitis are also known manifestations of glucagonoma [8,9]. A biopsy of the lesion is needed to confirm the diagnosis of NME.

Multiple endocrine neoplasia type 1

Multiple endocrine neoplasia type 1 (MEN1) is a disease that results from the inactivation of the menin tumor suppressor gene. It is inherited in an autosomal dominant manner. MEN1 is characterized by parathyroid adenomas, pancreatic islet tumors, and tumors of the anterior pituitary gland [10]. It has an estimated prevalence of 1-10 per 100,000 individuals [11].

Skin Manifestations

Cutaneous lesions are part of the diagnostic criteria for MEN1; two or more of these lesions are considered diagnostic, or one if there is a family history of MEN1 [12]. Angiofibromas, collagenomas, and lipomas are among the skin lesions that may occur in patients with MEN1 [13]. In a 1997 study concerning skin lesions in MEN1, angiofibroma was found to be the most common cutaneous manifestation with a prevalence of 88% of patients with MEN1 and was most often located on the face. These lesions were described as “telangiectatic, skin-colored, pink or light brown papules.” Collagenomas were present in 72% of patients and were described as “skin-colored to slightly hypopigmented, dome-shaped, well-circumscribed, firm, round to oval papules” and tend to occur on the upper portion of the torso, neck, and shoulders. Lipomas were present in 34% of patients and were located on the trunk, extremities, and scalp. Notably, *café-au-lait* macules were seen in 38% of the patients; however, they were not considered to be diagnostically significant [14]. Other manifestations included confetti-like hypopigmented macules (6%) and gingival papules (6%) [15].

Multiple endocrine neoplasia type 2

Multiple endocrine neoplasia type 2 (MEN2) occurs due to activating mutations in the RET proto-oncogene. Medullary carcinoma of the thyroid and pheochromocytomas are common in both MEN2A and MEN2B. In addition, parathyroid adenomas are common in MEN2A (10-15%), and mucosal neuromas are common in MEN2B [12]. The combined prevalence of MEN2A and MEN2B is approximately 1 per 35,000 individuals, with MEN2A representing 90-95% of the cases [16].

Skin Manifestations

MEN2A is associated with cutaneous lichen amyloidosis and is estimated to occur in 51% of the cases. These lesions tend to be located between the scapulae or on the extensor surfaces of the extremities. Pruritis is often the initial symptom, which is due to the deposition of an amyloid-like material. This leads to scratching of the affected areas, causing damage to keratinocytes. The damaged keratinocyte then degenerates and results in the characteristic scaly, pigmented, and papular skin lesions [17]. MEN2B is associated with mucosal neuromas. These present as verrucous papules and nodules [18]. They can be present on the inner eyelid, giving it a thickened appearance. They can also present on the lips, imparting an irregular appearance, as well as the anterior third of the tongue and buccal mucosa [19]. Additionally, patients with MEN2B can also have *café-au-lait* macules. The presence of dermal hyperneury, a cutaneous manifestation classically described in MEN2B syndrome, and multiple sclerotic fibromas has been reported as an associated clinical finding in a family of 11 individuals with familial medullary thyroid cancer-type of MEN2A syndrome [20].

Neurofibromatosis 1

Neurofibromatosis 1 (von Recklinghausen disease) is caused by a mutation in the neurofibromin gene, located at chromosome 17q11.2, leading to hyperfunction of the proto-oncogene RAS. It is transmitted in an autosomal-dominant manner with complete penetrance. Germline NF1 mutation in combination with the somatic mutation can cause complete loss of neurofibromin, as seen in pseudoarthroses and neurofibroma lesions [21,22]. Mosaicism can occur due to postzygotic somatic mutation (segmental NF1), classically resulting in unilateral cutaneous findings. For genetic analysis, the most accurate method is a multistep pathogenic variant detection protocol based on cDNA (mRNA) and genomic DNA sequences [23]. In addition to the below-mentioned dermatologic manifestations, Lisch nodules, or hamartomas of the iris, are common. Endocrine implications of NF1 can include growth hormone deficiency, growth hormone excess, precocious puberty, gynecomastia, and/or pheochromocytoma [24].

Skin Manifestations

Neurofibromas are the key dermatologic manifestation of NF1 and are present in >99% of patients. Schwann cells after losing the NF-1 allele secrete stem cell factors that drive mast cells into the lesion, which further express TGFβ leading to the proliferation of collagen and cellular (mast cells, Schwann cells, spindle cells, and vascular components) growth and tumor formation [23]. They can be subcategorized into cutaneous (focal or diffuse), subcutaneous, plexiform (nodular or diffuse), and spinal [25]. Most common cutaneous neurofibromas protrude through the skin and become pedunculated, while others are soft and palpable through the skin, while deeper lesions remain firm to the touch. The invagination on pressing the lesion (“Buttonhole” sign) is pathognomonic. Plexiform neurofibromas are specifically found among NF1 (30-50%) patients. These presentations follow a nerve fascicle, where the overlying skin can become rough and hyperpigmented [26]. These typically become noticeable during adolescence and their number may accelerate with age and pregnancy [27]. *Café-au-lait* macules, which are hyperpigmented, well-demarcated, round macules are also present in >99% of patients; these may manifest at birth and their number stabilizes with age such that more than six *café-au-lait* macules are highly suggestive of NF1. The number of the *café-au-lait* macules is not related to the severity of NF1 [28]. Freckling, presenting at three to four years of life as the small (1-4 mm), clustered pigmentary macules of the axillary region and groin (>85% of patients) [24], is nearly pathognomonic. These freckles, unlike the usual solar-induced freckling, almost exclusively develop in areas with minimal to no sun exposure and are believed to be due to increased friction, temperature, and/or moisture [27].

Von Hippel Lindau

Von Hippel Lindau (VHL) disease is associated with the mutation of the VHL tumor suppressor gene and is transmitted in an autosomal-dominant manner. Inadequate amounts of VHL protein (absent or decreased) fail to form the VHL-hypoxia-inducible factor (HIF)-ubiquitin complex, leading to rising HIF1A and HIF2A levels and uncontrolled angiogenic effects in patients [29]. The VHL mutation also leads to extracellular matrix abnormality, unstable control of cilia centrosome and microtubule, and cell cycle abnormalities [30,31]. Hemangioblastomas are the most prominent manifestation, occurring throughout the central nervous system, and tend to be infratentorial and multiple [32]. Renal cell carcinoma is common. Pancreatic neuroendocrine tumors, which are typically nonfunctional, can also occur. Pheochromocytoma may occur in 10-20% of affected patients [33].

Skin Manifestations

Skin manifestations associated with VHL disease are relatively uncommon [34]. When they do occur, they are generally nonspecific, including cutaneous hemangiomas and *café-au-lait* spots [35].

Pseudohypoaldosteronism

Pseudohypoaldosteronism is a syndrome characterized by resistance to the action of the steroid hormone aldosterone. Pseudohypoaldosteronism type I has two forms. The autosomal dominant form is caused by a mutation in the *NR3C2* gene coding for mineralocorticoid receptors specific to the kidney. The autosomal recessive form results from loss of activity in the epithelial sodium channel affecting all aldosterone targets, including the distal nephron. Pseudohypoaldosteronism type II can be autosomal dominant (frequently, *KLHL3 *mutations) or autosomal recessive. Mutations affecting *WNK4*, *WNK1*, and *CUL3 *genes or a heterozygous pathogenic variant or biallelic pathogenic variants in *KLHL3 *have been reported affecting the NaCl cotransporter in the distal tubule [36,37].

Skin Manifestations

Miliaria rubra, a pustular, erythematous skin lesion, has been described in infants with pseudohypoaldosteronism type I. These lesions are theorized to occur due to the obstruction of eccrine sweat glands caused by excess excretion of sodium that tends to occur during hyponatremic crises. The lesions improve with the correction of electrolyte abnormalities [38,39]. A white, opaque discharge around the eyes has also been reported [40].

Primary hyperparathyroidism

Primary hyperparathyroidism leads to hypercalcemia via overproduction of parathyroid hormone (PTH). Hypercalcemia occurs via increased production of 1,25-dihydroxy vitamin D, as well as increased osteoclast activity [41].

Skin Manifestations

Primary hyperparathyroidism does not commonly present with skin manifestations. Benign nodular calcification is a form of calcinosis cutis that may occur due to metastatic calcification in primary hyperparathyroidism. Skin lesions are characterized by indurated white papules or plaques located in the subcutaneous tissue. These tend to be located near the joints [41]. The most extreme skin manifestation of hyperparathyroidism is related to calciphylaxis, or calcification of blood vessels supplying cutaneous tissue. While this generally occurs in end-stage renal disease patients due to tertiary hyperparathyroidism, it may also occur as a result of primary hyperparathyroidism [42]. It begins as mottled skin lesions resembling livedo reticularis which then becomes painful and undergoes necrosis followed by the development of an overlying eschar. Sepsis may occur due to bacterial superinfection of these lesions. Gangrene of the digits or penis may also be a manifestation of this disorder [41].

Carney complex

Carney complex (CNC) is a rare genetic disorder characterized by pigmented lesions on the skin and mucosa, cardiac and noncardiac myxoma, and multiple endocrine and nonendocrine tumors [43]. It is associated with mutations in the *protein kinase A type I-alpha regulatory subunit *gene on chromosome 17q22-24 and is inherited in an autosomal-dominant manner, affecting half of all first-degree relatives.

Skin Manifestations

Dermatologic manifestations in patients with CNC vary from the more commonly present lentigines, seen in about 70-80% of cases, to blue nevi (small bluish domed papules with a smooth surface) and cutaneous myxomas. In addition, *café-au-lait* spots, depigmented lesions, and Spitz nevus have been reported [44,45]. Lentigines are small, brown-to-black macules, usually appearing before and during puberty anywhere on the body including the face, lips, genital area, or mucosal surface. These macules usually appear before and after puberty and gradually increase in number with age [46,47]. Epithelioid blue nevus is an interesting subtype of blue nevus that is very rare in the general population but is seen in patients with CNC. Patients present with intensive pigmentation and poorly circumscribed proliferative regions without dermal fibrosis [48]. Cutaneous myxomas are found in 30-55% of CNC patients and usually appear in the eyelid, external ear canal, and nipples [44]. These lesions can be localized in the dermis or the subcutaneous layer and are usually symptomless and less than 1 cm in diameter [49].

Pseudohypoparathyroidism

Pseudohypoparathyroidism (PHP) refers to a group of disorders characterized by inactivating mutations of *GNAS* (coding for Gs alpha unit of G protein-coupled receptor). Clinically, it leads to end-organ (kidney and bone) unresponsiveness to PTH. These syndromes could have either Albright’s hereditary osteodystrophy (AHO) phenotype or a normal phenotype. Patients with PHP often present with hypocalcemia, hyperphosphatemia, and elevated PTH.

Skin Manifestations

Short stature, obesity, brachydactyly, and subcutaneous calcifications are characteristic features of the AHO phenotype [50]. The skin can be dry, scaly, hyperkeratotic, and puffy in PHP. Additionally, there could be thinning of the skin and subcutaneous tissue. Nails can be opaque, brittle, and usually have a transverse ridge. There can be loss of scalp and body hair. Eczematous dermatitis, hyperkeratotic, and maculopapular eruptions have also been reported [1].

POEMS syndrome

POEMS syndrome (Polyradiculoneuropathy, Organomegaly, Endocrinopathy, Monoclonal plasma cell disorder, and Skin changes), also known as Takatsuki disease, is a rare paraneoplastic syndrome due to an underlying plasma cell disorder. The acronym refers to several, but not all, the features of this syndrome [51]. The increased levels of interleukin-6 (IL-6), IL-1β (IL-1β), and tumor necrosis factor-α, along with vascular endothelial growth factor, are involved in cellular proliferation and disease progression [52].

Skin Manifestations

Skin changes are present in 68-89% of the patients. The most common skin manifestations are hyperpigmentation and glomeruloid hemangioma. Hemangioma consists of dilated dermal vascular spaces with clusters of vessels resembling kidney glomeruli. Other cutaneous findings include hypertrichosis, dependent rubor, acrocyanosis, white nails, sclerodermoid changes, facial atrophy, flushing, cherry angiomata, and clubbing [53]. Paraneoplastic pemphigus has been linked to POEMS associated with Castleman disease and is recognized as a bad prognostic factor [54].

Cowden syndrome

Cowden syndrome is an autosomal-dominant disorder associated with an increased risk of both benign and malignant tumors originating from the derivatives of all three germ cell layers. The syndrome is caused by a mutation in the tumor suppressor *PTEN*. Patients with Cowden syndrome appear to be particularly disposed to breast cancer, thyroid cancer, and endometrial cancer, although various other types of tumors have also been observed [55]. The estimated prevalence of Cowden syndrome is approximately 1 in 200,000 to 250,000 [56].

Skin Manifestations

The mucocutaneous manifestations are often the initial findings that aid in the diagnosis. The vast majority of patients (99% by the third decade of life) develop hamartomas (benign growths), generally found on the skin and mucous membranes. These include trichilemmomas, papillomatous papules, acral keratoses, and palmoplantar keratoses. Trichilemmomas are hamartomas derived from the infundibulum (outer root sheath) of the hair follicle. These are usually found on the face. Papillomatous papules are benign neoplasms of the epithelium. Extensive oral mucosal papillomatosis or numerous conjoined papillomas causing a cobblestoned appearance is common [55,57].

Hypopituitarism

Hypopituitarism is a deficiency of one or all anterior pituitary hormones (follicle-stimulating hormone [FSH], luteinizing hormone [LH], adrenocorticotropic hormone [ACTH], thyroid-stimulating hormone [TSH], prolactin, growth hormone). There are numerous etiologies of hypopituitarism including pituitary adenoma, history of pituitary surgery/radiation therapy, granulomatous disease, or traumatic brain injury [58].

Skin Manifestations

Skin findings in hypopituitarism depend on which hormones are deficient. Reduced FSH/LH causes secondary hypogonadism. In women, this can result in decreased breast tissue. In men, this is often seen clinically as gynecomastia, decreased axillary and pubic hair, and decreased muscle mass [59]. Reduced ACTH does not cause any specific cutaneous finding. Reduced TSH can result in symptoms of hypothyroidism including dry skin and facial edema. Reduced prolactin does not generally cause any dermatologic manifestations. Reduced sweat secretion is noted with reduced skin thickness and increased dryness [60].

Cushing’s syndrome

Cushing’s syndrome (CS) is characterized by a constellation of clinical features resulting from chronic glucocorticoid excess. Excess cortisol can either be ACTH-dependent or ACTH-independent. Based on the underlying source, CS can be a result of (1) pituitary hypersecretion of ACTH (Cushing disease), (2) ectopic secretion of ACTH by nonpituitary tumors, and (3) excess secretion of cortisol from the adrenal glands [1].

Skin Manifestations

The most common feature of glucocorticoid excess includes central adiposity involving the face, neck, trunk, and abdomen resulting in a typical cushingoid appearance. Fat deposits in the cheeks result in a classical facial plethora (moon facies) and are accompanied by often accompanied by accumulation of subcutaneous fat in the dorsocervical region (buffalo hump). Excess cortisol inhibits collagen synthesis resulting in thinning of the skin and easy bruising after minimal trauma. Patients with CS may also have purple (violaceous) and wide (>1 cm in diameter) striae on the abdomen and lower flanks, which is considered a pathognomonic sign of CS. In addition, hyperpigmentation can also be seen in patients with chronic ACTH-dependent CS (more common ectopic ACTH syndrome than in patients with pituitary hypersecretion of ACTH) [1]. Other cutaneous manifestations include acanthosis nigricans due to insulin resistance.

Primary adrenal insufficiency

Primary adrenal insufficiency is a condition characterized by failure of the adrenal glands to produce cortisol. This is most commonly caused by Addison’s disease, an autoimmune adrenalitis [61].

Skin Manifestations

Hyperpigmentation of the skin and mucous membranes is the main dermatologic finding in Addison’s disease, occurring in >90% of patients. It occurs secondary to cortisol deficiency leading to increased ACTH and melanocyte-stimulating hormone which increases melanin synthesis [62]. Hyperpigmentation mainly occurs in sun-exposed areas as well as areas with increased friction. Patients can present with darkening of hair and nails. Hyperpigmentation can resolve with adequate replacement with steroids. There is an increased risk of other autoimmune conditions in patients with Addison’s disease, including vitiligo which occurs in about 10% of patients [63].

Waterhouse-Friderichsen syndrome

Waterhouse-Friderichsen syndrome (WFS) is adrenal hemorrhage (usually bilateral) caused by severe bacterial infection. It is generally associated with meningococcemia, although numerous other bacteria have also been implicated.

Skin Manifestations

The primary dermatologic finding of WFS is the presence of petechial eruptions on the trunk, which can be seen in up to 75% of cases. The petechiae often progress to purpuric rash [64].

Polyglandular autoimmune syndrome type 1

Polyglandular autoimmune syndrome type 1, also known as autoimmune polyendocrinopathy-candidiasis-ectodermal dystrophy (APECED), is a rare immunodeficiency syndrome due to mutation of the *autoimmune regulator* gene (*AIRE*). It is characterized by chronic mucocutaneous candidiasis (CMC), hypoparathyroidism, adrenal insufficiency, and primary hypogonadism [65].

Skin Manifestations

A characteristic feature of APECED is CMC [66]. This is generally the first symptom of the disorder to become apparent (in about 60% of patients), often in early childhood. Candidiasis affects all patients by the age of 40 and can be oral, gastrointestinal, or genitourinary. Hyperpigmentation can be seen among patients with adrenal insufficiency, although adrenal insufficiency is only present in about 5% of the cases. Additionally, people with APECED often have accompanying autoimmune disorders which may include dental enamel hypoplasia, keratoconjunctivitis, alopecia, or vitiligo [65].

Adrenoleukodystrophy

X-linked adrenoleukodystrophy (X-ALD) is the most common peroxisomal disorder with heterogeneous clinical presentations. It is caused by mutations of the *ABCD1* gene, which is a peroxisome transporter and prevents normal transport and breakdown of very-long-chain fatty acids leading to their accumulation in affected organs, particularly the central nervous system, Leydig cells, and adrenal cortex. There are at least six distinct types of X-ALD, including childhood cerebral forms, adrenomyeloneuropathy, and Addison’s disease alone [67].

Skin Manifestations

Many patients with X-ALD have hyperpigmentation secondary to adrenal insufficiency and increased ACTH. Adult ALD can also cause thinning of scalp hair [67].

Extreme insulin resistance syndrome

There are several inherited severe insulin resistance syndromes, namely, Donohue syndrome (leprechaunism, the most severe), Rabson Mendenhall syndrome (intermediate severity), and type A insulin resistance syndrome (least severe).

Skin Manifestations

The most common dermatologic finding is acanthosis nigricans, characterized by thickening, hyperpigmentation, and velvet-like appearance usually on the neck, axilla, and groin. Less commonly, acanthosis nigricans can also be associated with gastrointestinal adenocarcinomas [68].

Diabetes mellitus

Diabetes is the most common endocrine disorder globally and is characterized by hyperglycemia over an extended period of time. The two most common subtypes are type 1 and type 2 diabetes mellitus.

Skin Manifestations

Different cutaneous manifestations, some of which are specific and others nonspecific (related to metabolic changes), can be seen in patients with diabetes. These include recurrent cutaneous fungal or bacterial infections, necrobiosis lipoidica diabeticorum (0.3-1.6%), diabetic bullae (0.5%), and autoimmune-related cutaneous disorders (such as vitiligo). Diabetic dermopathy is the most common dermatological finding, occurring in up to 70% of adult patients with diabetes [1,69].

Table 1 presents a summary of cutaneous manifestations seen in endocrine disorders.

**Table 1:** Summary of cutaneous manifestations seen in endocrine disorders. MEN1: multiple endocrine neoplasia type 1; MEN2: multiple endocrine neoplasia type 2; NF1: neurofibromatosis 1; POEMS: Polyradiculoneuropathy, Organomegaly, Endocrinopathy, Monoclonal plasma cell disorder, and Skin changes; NLD: necrobiosis lipoidica diabeticorum

Endocrine disorder	Key skin lesion	Incidence/Prevalence	Sites of predilection	Other manifestations
Graves’ disease [1-3]	Thyroid dermopathy	5% in patients with Graves’ disease and 15% in patients with Graves’ disease and orbitopathy	Pretibial area, lower extremity, toes, and areas of trauma	Warm, moist, and thin skin, onycholysis (Plummer’s nails), thinning of hair
Hashimoto’s thyroiditis [1,5]	Nonpitting edema	Common	Pretibial area	Cold dry skin, diffuse hair loss, macroglossia, chronic urticaria, carotenemia
Glucagonoma [8,9]	Necrolytic migratory erythema	Seen in all patients	Groin, intertriginous areas, extremities, thighs, buttocks, perioral area	Angular cheilitis/stomatitis
MEN1 [10,11,13]	Facial angiofibroma	80–90%	Face	Telangiectatic, skin-colored, pink or light brown papules, nodules, *café-au-lait* macules
	Collagenomas	70–80%	Upper torso, neck, shoulders	
	Lipoma	30–35%	Trunk, extremities, scalp	
MEN2A [12,16]	Lichen amyloidosis	50%	Scapulae, extensor surfaces, extremities	Pruritis
MEN2B [16]	Mucosal neuromas	Almost all	Eyelids, lips, tongue	*Café-au-lait* macules
NF1 [21-24,27,28]	Neurofibromas	>99%	Mainly abdomen, chest, and spine	Rough and hyperpigmented skin
	Café-au-lait	>99%	Any location	
	Freckling	>85%	Axillary, groin	
Von Hippel Lindau syndrome [29-35]	Cutaneous hemangiomas, *café-au-lait* spots	Uncommon	Mainly trunk and extremities	Capillary malformation
Pseudohypoaldosteronism [36-40]	Miliaria rubra	Common in a hot humid environment	Face, arms, trunk	Whitish periocular discharge
Primary hyperparathyroidism [41,42]	Calcinosis Cutis	Uncommon	Joints	Indurated white papules or plaques, eschar, gangrene, eschar
	Calciphylaxis	Uncommon	Genital area	
Carney complex [43-49]	Lentigines blue nevi and cutaneous myxomas	70–80%, 30–55%	Face, lips, genital area, mucosal surface, eyelid, external ear canal, and nipples	Blue nevi, intensive pigmentation
Pseudohypoparathyroidism [1,50]	Eczematous dermatitis	Uncommon	Nails, hair, skin	Coarse and sparse hair
POEMS syndrome [51-54]	Hemangioma and hyperpigmentation	68–89%	Nails, face	Skin thickening, hypertrichosis, white nails, and acquired facial lipoatrophy
Cowden syndrome [55-57]	Hamartomas	99%	Skin, mucous membranes, face	Cobblestoned appearance
Hypopituitarism [58-60]	Hypogonadism, hypothyroidism, secondary adrenal insufficiency	Depending on which hormones are deficient	Muscles, axillary, and public hair in men with hypogonadism, whereas breast tissue and genitourinary tract in women	Dry skin, facial edema, short stature in children, increased fat mass
Cushing’s syndrome [1]	Central adiposity, moon facies, dorsocervical hump, easy bruising, thinning of the skin, purple and wide striae	Variable based on the severity of disease	Abdomen and lower flanks	Acanthosis nigricans
Primary adrenal insufficiency [61-63]	Hyperpigmentation	>90%	Skin, mucous membranes, hair, nails	Vitiligo
Waterhouse-Friderichsen syndrome [64]	Small skin macule and papule that progress to petechial eruption then large coalescent plaque	75%	Trunk	Purpuric rash
Polyglandular autoimmune syndrome type 1 [65,66]	Chronic mucocutaneous candidiasis	60%	Oral, gastrointestinal tract, genitourinary tract	Hyperpigmentation, dental enamel erosion, alopecia, vitiligo
Adrenoleukodystrophy [67]	Hyperpigmentation	Common	Scalp	Thinning of scalp hair
Extreme insulin resistance syndromes [68]	Acanthosis nigricans	Common	Neck, axilla, groin	Gastrointestinal adenocarcinomas
Diabetes mellitus [1,69]	Acanthosis nigricans	Up to 70% of adult patients	Anterior shin, back of the neck	Cutaneous fungal and bacterial infection, NLD, diabetic bullae, and autoimmune skin diseases

## Conclusions

Hormone excess or deficiency often results in skin manifestations, varying from simple superficial skin lesions to more complex lesions. While few dermatological manifestations can be pathognomonic of the underlying endocrine disorders (e.g., NME in patients with glucagonoma), the majority of these dermatological manifestations are nonspecific and require more careful evaluation. Some of these skin lesions are already known dermatologic diseases with an increased frequency of occurrence in patients with endocrine disorders. Furthermore, in some endocrine conditions, cutaneous lesions are part of the diagnostic criteria (e.g., MEN1). Moreover, these dermatological manifestations can either be localized lesions (e.g., facial angiofibroma in MEN1) or can be located on a widespread area of the skin (e.g., VHL). Hence, skin can be considered to be a screen displaying underlying endocrine disorders, either due to hormone excess or deficiency. In this narrative review, we summarized various endocrine conditions and their associated skin disorders. Healthcare providers should be able to recognize these disorders, associate them with the underlying endocrine problem, and perform further workup to make a final diagnosis.
